# Perception of Musical Emotion in the Students with Cognitive and Acquired Hearing Loss

**Published:** 2018

**Authors:** Malihe MAZAHERYAZDI, Mina AGHASOLEIMANI, Maryam KARIMI, Pirooz ARJMAND

**Affiliations:** 1 Department of Audiology, School of Rehabilitation Sciences, Iran University of Medical Sciences, Tehran, Iran; 2 Department of Audiology, School of Rehabilitation Sciences, Shahid Beheshti University of Medical Sciences, Tehran, Iran; 3 Department of Traditional and Ancient Art, Faculty of Art and Architecture, Shahid Bahonar University, Kerman, Iran

**Keywords:** Deafness, Children, Acquired hearing loss, Emotion, Music

## Abstract

**Objective:**

Hearing loss can affect the perception of emotional reaction to the music. The present study investigated whether the students with congenital hearing loss exposed to the deaf culture, percept the same emotion from the music as students with acquired hearing loss.

**Materials & Methods:**

Participants were divided into two groups; 30 students with bilaterally congenital moderate to severe hearing loss that were selected from deaf schools located in Tehran, Iran and 30 students with an acquired hearing loss with the same degree of hearing loss selected from Amiralam Hospital, Tehran, Iran and compared with the group of 30 age and gender-matched normal hearing subjects served our control in 2012. The musical stimuli consisted of three different sequences of music, (sadness, happiness, and fear) each with the duration of 60 sec. The students were asked to point to the lists of words that best matched with their emotions.

**Results:**

Emotional perception of sadness, happiness, and fear in congenital hearing loss children was significantly poorly than acquired hearing loss and normal hearing group (*P*<0.001). There was no significant difference in the emotional perception of sadness, happiness, and fear among the group of acquired hearing loss and normal hearing group (*P*=0.75), (*P*=1) and (*P*=0.16) respectively.

**Conclusion:**

Neural plasticity induced by hearing assistant devises may be affected by the time when a hearing aid was first fitted and how the auditory system responds to the reintroduction of certain sounds via amplification. Therefore, children who experienced auditory input of different sound patterns in their early childhood will show more perceptual flexibility in different situations than the children with congenital hearing loss and Deaf culture

## Introduction

The music has always been considered to sound either happy or sad. Music has been though as a propellant of intense emotion. However, there are discrepancies in the literature about how the music can actually evoke emotions. The related emotions consist of a subjective feeling; however, others expand the definition to contain other aspects, such as cognition, physiologically arousal state motor and behavioral expressions, too. 

Two theories have been delineated about the possible links in between music and emotion, emotivist and cognitivist. Music can actually evoke or induce feeling in listeners. By contrast, some suggest that listeners simply feel positive or negative when they like or dislike the music. Listeners characterize the music as being happy or sad, originating from the fact that the music expresses happiness or sadness (1). . Perception of the emotional cues in social relations,is an important part of normal social development and communication. So music as a nonverbal tool may play an important role in managing of different social skills during development (2). Children with normal hearing react to the music, spontaneously and effortlessly and often have an exciting emotional response (3). As the normal hearing children grow, even ones as young as 3 yr old, can make a link between musical inputs and specific emotion. Different studies pointed that the children similar to adult could judge labels same as happy, sad or angry for classical music segments (4-6). Music, like language, is a typical human feature. Music perception emerges spontaneously in somewhat specified time, in just quite early developmental stage. In line with appearance of early singing abilities, during the first month of life, infants start vocalization, which can be the precursors of music and speech perception (7). Unfortunately, deprivation of hearing sense and deficient of pitch perception, especially in congenitally deaf children can affect achievement of both perception and production of speech; additionally, skills such as processing of musical aspects and emotional prosody like pitch range, direct and rhythmicity will be affected, too. 

Emotional prosody includes semantic information (differences between statement and question) and affective intent (differences between happiness and sadness) is crucial for achieving the meaning of speech and music (8). Deaf children have problems in production of appropriate rate, loudness, stress, and their laryngeal and resonance quality of expressive prosody were defaced. Moreover, they prefer to use vision rather than hearing in situation where both of the modalities are affected (8). 

There is a relation between music and emotion. Some variables such as cultural circumstances may play a role in the judgment of emotional perception of music. On the other hands, some dimensions of music such as timbre and pitch may influence the listener’s perception of emotion of music. Neural structures that participate in the processing of auditory information are the other elements that incorporated in perceiving of the musically induced emotion. Any lesion in auditory system can disturb the perception of emotional reaction to the music. Deaf children were compared (those from a state school dedicated to the deaf where the deaf culture was supported strongly) to age-matched children who attended public schools. Deaf students had a different reaction to musically induced emotion. The authors presented the following facts for the justification of their finding: 1- In spite of using amplification, deaf students may not have accessibility to all auditory details that lead to the leakage of some auditory experiences. 2- Social environment has an important role in perceiving the specific emotion; there is little or no attention to give to sound in deaf school. 3- Familiarity with the type of presented music is an effective factor in recognizing emotion, so musical training can be a practical factor in perceiving the emotional content of music. Culture and experiences can affect the perception of music and suggested that musical therapy can increase the musical experiences of children within the same culture (5). 

Children, who experienced auditory input of different sound patterns in their early childhood, will show more perceptual flexibility in different situations. Thus, the purpose of the present study was to investigate whether the students with congenital hearing loss exposed to the deaf culture, percept the same emotion from the music as students with acquired hearing loss.

## Materials & Methods

The study participants were 60 students divided into two groups. The first group consisted of 30 students with bilaterally congenital moderate to severe hearing loss who were studying in deaf cultured high school from deaf schools and Amiralam Hospital Tehran, Iran in 2012. Mean (± standard deviation) of age was 16.77 (±1.25) yr and range, 15 – 19 yr, 16 boys, and 14 girls. Overall, 30 students with an acquired hearing loss of the same degree, who attending audiology clinic in a university hospital center participated as a second group. Mean (± SD) of age was 22.20 (± 1.32) yr and range, 20-25 yr 14 girls and 16 boys. A group of 30 age and gender-matched normal hearing subjects served our control group. Mean (± SD) of age was 19.83 (± 2.42) yr and range, 16-28 yr), 15 girls and 15 boys. Inclusion criteria were 1) the student with 15-30 yr old, 2) moderate to severe sensorineural hearing loss in each group of children with hearing loss, and 3) hearing threshold ≤ 25 dB in control group. Individuals with hearing loss (either congenital or acquired) were all fitted by bilateral hearing aids.

**Table 1:** Relation of three variables between three groups, congenital with acquired, congenital with normal and Acquired with normal

	Sadness		Happiness		Fear	
Between groups	Mean difference (±sd )	*P* value	Mean difference (±sd	*P* value	Mean difference (±sd	*P* value
Congenital with acquired	0.366(±0.507)	0.001	0.333(±0.479)	0.001	0.366(±0.449)	0.002
Congenital with normal	-0.433(±0.305)	0.001	0.333(±0.000)	0.001	0.666(±0.490)	0.001
Acquired with normal	-0.066(±0.182)	0.751	0.333(±0.000)	1.00	0.300(±0.253)	0.016

**Table 2:** Relation of three variable and sex

Variables	Groups	Mean(±sd)	T value	Degrees of freedom	*P*-value
Sadness	Boy	0.777(±0.420)	0.522	88	0.603
	Girl	0.822(±0.386)			
Happiness	Boy	0.844(±0.366)	1.34	88	0.184
	Girl	0.933(±0.252)			
Fear	Boy	0.577(±0.499)	0.643	88	0.522
	Girl	0.644(±0.484)			

**Musical stimuli**

The musical stimuli used in this study consisted of three different sequences of music, each with the duration of 60 sec randomly ordered to depict one of the three basic emotions – happiness, sadness, and fear. We incorporated those musical pieces clearly intended their meaning by their composers. 

**Procedure**

Participants were tested in a typical silent room. Individuals with hearing loss took their hearing aids off. They were seated in front of a table, there were three lists of words on the table, and each could express happiness, sadness or fear. Then they listened to the piece of music through the TDH39 headphone at their most comfortable level (MCL). After listening to each excerpt, they were asked to point to the lists of words that best matched with their emotions.

**Statistical analysis**

SPSS software (ver. 21 Chicago, IL, USA) was used for statistical evaluation. The criterion for statistical significance was defined as *P-*value≤0.05. Kolmogorov-Smirnov test was used to determine the normal distribution of variables. To compare different values between study and control groups, one-way ANOVA analysis variance test was used. 

## Results

The variables of happiness, sadness and fear did not have statistically significant differences (*P*>0.05). There was a significant difference between groups considering their performance on the variables of happiness, sadness, and fear; children with congenital hearing loss performed poorer than those with acquired hearing loss and control group. Emotional perception of sadness in congenital hearing loss children was significantly poorly than acquired hearing loss and normal hearing group (df=2, F=12/763, *P*<0.001). There was no significant difference in the emotional perception of sadness between the group of acquired hearing loss and normal hearing group (*P*=0.751). Additionally, perception of happiness and fear was significantly different between congenital and acquired hearing loss children (df =2, F=14/50, *P*<0.001) and (df=2, F=19/79, *P*<0.001) for happiness and fear, respectively. However, there were no significant differences between the group of acquired hearing loss and normal hearing group considering the perception of happiness and fear (Table 1). The results of each of three emotional perception variables in two genders are presented in Table 2. There were no significant differences in happiness, sadness and fear perception between two genders, meaning that both groups behaved the same in the emotional perception.

No significant correlation was found between age and the overall test scores on none of three emotional variables, r= -0.054, *P*=0.615 for sadness variable, r= -0.022,* P*=0.834, for happiness and r= -0.044, *P*=0.973 for the fear perception.

## Discussion

The purpose of the present study was to investigate whether children with congenital hearing loss with deaf culture had the same emotional reaction to music as children with acquired hearing loss or not. They do not show the same emotion to the music. There were significant differences between two groups considering musical perception and more mistakes were seen in children with congenital hearing loss. The children with congenital hearing loss may not listen to the music as well as acquired hearing loss, in spite of receiving suitable hearing aid prescription according to their audiograms. Listening is a complex task and needs higher level of central auditory processing rather than hearing (9). Cognitive influences such as attention may also play an important role in emotional perception especially when there are two talkers rather than one because the listeners must follow what is being said and without appropriate experimental control they may suffer from immaturity of sensory function (3, 10). In the auditory system, children with congenital hearing loss are more susceptible to auditory distractions and more inattentive about their surrounding environment. In spite of sound detection ability immediately after birth; some auditory aspects such as sound localization and temporal processing depend on maturation of the central auditory system to adolescence (11). If children with acquired hearing loss receive, enhanced auditory experience in critical periods it results in shaping the auditory brain function that has an important role in speech processing, cognition, and emotional prosody. Perception of musical emotion in deaf students was investigated. Results indicated a significant difference between the deaf and normal hearing participants that was in accordance with our study results. Deaf children were less aware of musical style and most of them received actually no musical training (5). 

Cochlear implant (CI) as one of the rehabilitation approach, enable many deaf children to achieve more appropriate speech perception and perhaps enjoy the music. However, situation is very different for music perception, because with most CI signal processing strategies, pitch information may be transmitted insufficiently (12). The musical input that implant users receive is different from the music experienced by normal-hearing ones. The limitation of CI devices can potentially alter the processing of auditory information. For example, those who received unilateral CI, suffer from inaccurate representation of fine temporal cues and lower speech resolution compared to those implanted bilaterally (8). A study verified whether children who use cochlear implant can identify the emotional content carried in music. The authors studied 7-13 yr old children with mean duration of 2.9 yr of CI usage. The children were asked to judge about 32 music pieces to determine whether they sound happy or sad by pointing to the picture of a smiling or sad face. Children with CIs had a poor performance on this task than their normal-hearing peers; however, they were able to perceive emotional content of music (2). 

In line with our research work, some studies have been encountered in the literature addressing emotional prosody perception in those with hearing loss. In a study (8), hearing abilities of prelingually deaf children of 7-13 yr old were compared with age and gender-matched normal hearing ones, the speech prosody perception and facial affect were evaluated. In the emotional perception task, participants were presented with one of four emotion words (happy, sad, angry, or fearful) and they should point to the appropriate written word. Then the same emotion words were repeated and the participants should point to the appropriate face image. The authors reached to the conclusion that children using CI, performed weaker than control group on the speech prosody perception task (*P*<0.001). In accordance with our findings, children using CI, could identify and repeat words correctly in both closed and open set tests, meaning that they had an access to the acoustic cues available in these words. They knew how to use this information to identify words presently acoustically, but these children had problems in perceiving an emotion presented by prosody in speech (8). In addition, under the developmental viewpoint, perception of happiness emotion recognized first and then sadness, anger and then fear and surprise (8, 13). Children with CI understand the spoken words correctly, but they could not identify the emotion of what is said. Their explanation was as follows: 1- the device may have a limitation in processing the relevant information necessary for emotional prosody perception, such as inaccurate representation of fine temporal cues and lower speech resolution. 2- Children with CI may not develop normal cortical representation of speech prosody cues due to the hearing loss, especially when the right hemisphere, which is responsible for expressing and recognizing of speech prosody, was affected (8). 

Some studies dealt with temporal resolution skills in those with cochlear hearing loss, which use cochlear implant. Their results showed abnormality in the frequency resolution skills, which lead to an abnormal music perception; because the accurate perception of music requires the precious discrimination of frequency modulations. The improvements in speech recognition by usage of a hearing devise overtime may not necessary result in improvement of music perception (14, 15). Sensorineural hearing loss has a profound impact on the integrity of auditory system, affecting both peripheral and central auditory processing. For example, deprivation due to the hearing loss can affect auditory subcortical neural timing, which leads to the widening of the auditory ﬁlters and reduction of precious spectral information encoding (16). Neural plasticity induced by hearing aid usage, may be affected by the following facts: 1- the time when a hearing aid was first fitted, 2- how the auditory system respond to the reintroduction of certain sounds via amplification (17). 

The findings of the present study are in line with previous studies, where found no age and gender effects on musical perception. (5, 18, 19). Culture and auditory experience can affect the perception of musical emotion. Darrow states that discussion about the emotional aspects of music can also be extended to stimulate the thoughts and feelings that deaf children may experience; additionally, we cannot change the deaf culture, but with incorporating the music, we can increase musical experience of children who received deaf culture (5).

**In Conclusion, **perception of musical emotion is depended on auditory experience and culture of someone. So the usage of amplification that introduce high resolution and fine temporal cues as soone as possible in children, can affect development and integration in central nervouse system 
